# Prevalence of Treatment‐Resistant Schizophrenia: A Nationwide Multicenter Study

**DOI:** 10.1002/npr2.70163

**Published:** 2026-09-13

**Authors:** Yusuke Arai, Toru Horinouchi, Hiroyuki Muraoka, Junya Matsumoto, Kazuhiko Yamamuro, Toshinori Nakamura, Hisashi Yamada, Toshiaki Onitsuka, Yuka Yasuda, Naoki Hashimoto, Kazutaka Ohi, Shinichiro Ochi, Yasushi Kawamata, Keisuke Mori, Hirotaka Yamagata, Takashi Tsuboi, Fumitoshi Kodaka, Jun‐ichi Iga, Shusuke Numata, Ken Inada, Koichiro Watanabe, Hikaru Hori, Norio Yasui‐Furukori, Ryota Hashimoto

**Affiliations:** ^1^ Department of Community Mental Health Shinshu University School of Medicine Matsumoto Nagano Japan; ^2^ Department of Psychiatry and Neurology Hokkaido University Hospital Sapporo Hokkaido Japan; ^3^ Department of Psychiatry Kitasato University, School of Medicine Sagamihara Kanagawa Japan; ^4^ Department of Pathology of Mental Diseases National Institute of Mental Health, National Center of Neurology and Psychiatry Kodaira Tokyo Japan; ^5^ Center for Health Control Nara Medical University Kashihara Nara Japan; ^6^ Department of Psychiatry Nara Medical University School of Medicine Kashihara Nara Japan; ^7^ Department of Psychiatry Shinshu University School of Medicine Matsumoto Nagano Japan; ^8^ Department of Neuropsychiatry Hyogo Medical University Nishinomiya Hyogo Japan; ^9^ NHO Sakakibara National Hospital Tsu Mie Japan; ^10^ Life Grow Brilliant Mental Clinic, Medical Corporation Foster Osaka Osaka Japan; ^11^ Department of Psychiatry Gifu University Graduate School of Medicine Gifu Gifu Japan; ^12^ Department of Neuropsychiatry Ehime University Graduate School of Medicine Toon Ehime Japan; ^13^ Department of Psychiatry Dokkyo Medical University School of Medicine Mibu Tochigi Japan; ^14^ Department of Psychiatry The Jikei University School of Medicine Minato‐ku Tokyo Japan; ^15^ Department of Neuropsychiatry Kyorin University School of Medicine Mitaka Tokyo Japan; ^16^ Department of Psychiatry, Graduate School of Biomedical Science Tokushima University Tokushima Tokushima Japan; ^17^ Department of Psychiatry, Faculty of Medicine Fukuoka University Fukuoka Fukuoka Japan

**Keywords:** clozapine, prevalence, schizophrenia, treatment‐resistant schizophrenia

## Abstract

**Background:**

Schizophrenia is characterized by positive symptoms, negative symptoms, and cognitive impairment and is primarily treated with antipsychotic medications. However, a subset of patients responds inadequately to treatment and is classified as having treatment‐resistant schizophrenia (TRS). The prevalence of TRS among patients with schizophrenia remains insufficiently characterized. This study aimed to determine the prevalence of TRS using nationwide data and standardized diagnostic criteria.

**Methods:**

This study included patients with schizophrenia who were discharged between 2016 and 2024 from 205 institutions participating in the Effectiveness of Guidelines for Dissemination and Education in Psychiatric Treatment (EGUIDE) project and authorized to prescribe clozapine. TRS was defined according to standardized diagnostic criteria used in Japan. The prevalence of TRS was calculated among patients who underwent a TRS examination by their treating psychiatrist.

**Results:**

Among 18 914 patients, 9870 (52.2%) underwent a TRS examination. Among these 9870 patients, 2819 were classified as having TRS, corresponding to a prevalence of 28.6%. Among the 2819 patients with TRS, 1307 were prescribed clozapine at discharge, corresponding to a clozapine treatment rate of 46.4%.

**Conclusions:**

This study had the largest sample size among single‐cohort studies investigating the prevalence of TRS. The prevalence of TRS was 28.6% among patients with schizophrenia who underwent a TRS examination and was nearly identical to the pooled prevalence reported in studies at low risk of bias in a previous meta‐analysis. These findings may provide important evidence regarding the prevalence of TRS in routine clinical practice.

## Abstract 2

Overview of the study design and main findings. Among 18 914 patients with schizophrenia discharged from 205 GUIDE‐participating institutions in Japan between 2016 and 2024, 9870 (52.2%) underwent a TRS examination. The prevalence of treatment‐resistant schizophrenia (TRS) was 28.6% (2819/9870). Among patients with TRS, 1307 (46.4%) were prescribed clozapine at discharge.

## Graphical abstract

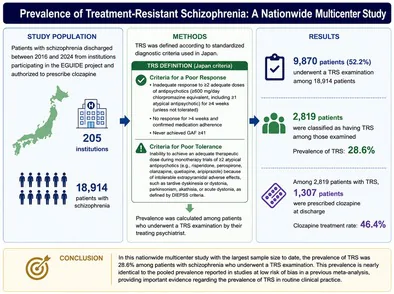

## 1 Introduction

Schizophrenia is a psychiatric disorder characterized by both positive and negative symptoms and cognitive impairment that affects approximately 0.4% of the global population [1]. Schizophrenia is also associated with marked impairments in social and occupational functioning, resulting in a substantial burden on affected individuals and society [2]. Antipsychotic medications are effective at improving positive symptoms and preventing relapse [3]; however, a subset of patients show an insufficient treatment response and are defined as having treatment‐resistant schizophrenia (TRS). Clozapine is the only antipsychotic with established efficacy for treating TRS [4], underscoring the importance of its timely initiation in patients with TRS. The definition of TRS varies across clinical guidelines, and recent systematic reviews consistently identify the core feature of TRS as an inadequate treatment response despite trials of at least two different antipsychotics administered at adequate doses and for sufficient durations [5]. However, the diagnostic criteria for TRS vary across countries and regions, with some definitions encompassing not only an inadequate response to antipsychotic treatment but also intolerance to antipsychotic medications [6]. Given this background, a meta‐analysis of 50 studies estimated the prevalence of TRS to be 36.7% [7]. However, the prevalence reported in the individual studies included in the meta‐analysis varied widely, ranging from 15.4% to 75.6%, indicating substantial heterogeneity. Thus, the prevalence of TRS remains insufficiently characterized. Moreover, no previous study has investigated the prevalence of TRS using standardized Japanese diagnostic criteria. Therefore, the present study aimed to determine the prevalence of TRS using a large, nationwide, multicenter dataset derived from routine clinical practice in Japan.

## 2 Methods

This study was conducted as part of the Effectiveness of Guidelines for Dissemination and Education in Psychiatric Treatment (EGUIDE) project. The EGUIDE project is a nationwide, prospective educational research program designed to promote the dissemination and implementation of treatment guidelines for patients with schizophrenia and major depressive disorder (MDD) [8]. In the schizophrenia component of the program, the participating psychiatrists were trained in the standardized Japanese diagnostic criteria for the TRS [6]. They were instructed to conduct a TRS examination in accordance with these criteria and to document the results in the discharge summaries. To date, the EGUIDE project has generated numerous studies on clinical practice patterns and guideline adherence in patients with schizophrenia and MDD, beyond those specifically addressing TRS [9, 10, 11, 12, 13, 14, 15, 16, 17, 18, 19, 20]. This study used data collected annually from participating institutions across Japan through the EGUIDE project between 2016 and 2024. The study included patients with schizophrenia who had been hospitalized for at least 4 days and subsequently discharged from one of 205 EGUIDE‐participating institutions that met the accreditation criteria for clozapine‐prescribing facilities (Table S1). The variables analyzed in this study were age, sex, type of institution, whether a TRS examination was conducted, the result of the TRS examination (TRS or non‐TRS), and clozapine prescription status at discharge among patients with TRS. Data were collected at each institution using an opt‐out consent procedure. This study was approved by the Ethics Committee of the National Center of Neurology and Psychiatry (B2022‐004) and the Ethics Committee of Dokkyo Medical University Hospital (R‐98‐4 J).

We defined the examination rate of TRS on the basis of the record that was submitted, as reported previously [6]. We checked the types and dosages of all the psychotropic drugs, including clozapine, and examined each patient for TRS. This study applied the definition of TRS by the Clozapine Appropriate Use Committee in Japan, which is included in the insert package (Table S2) [21]. Briefly, TRS is defined as a poor response or poor tolerability.

First, the proportion of patients who underwent a TRS examination was calculated for the overall study population. Background characteristics were then compared between patients who did and did not undergo a TRS examination. Age was compared using Student's *t*‐test, whereas sex and type of institution were compared using the chi‐square test. Bonferroni correction was applied to account for the three comparisons, and a corrected significance threshold of *p* < 0.0167 was used. Because TRS status could be determined only among patients who underwent a TRS examination, the prevalence of TRS was calculated in this subgroup. Finally, the clozapine treatment rate among patients with TRS was calculated using the number of patients classified as having TRS as the denominator and the number of those prescribed clozapine at discharge as the numerator.

## 3 Results

The demographic information of hospitalized patients with schizophrenia and the prevalence of TRS are shown in Table 1. Among the 18 914 patients included in the study, 9870 (52.2%) underwent a TRS examination, whereas 9044 (47.8%) did not. The mean age was 46.1 ± 15.6 years in the TRS examination group and 47.2 ± 16.0 years in the non‐examination group, with a statistically significant between‐group difference (*p* = 1.1 × 10^−6^). The distribution of institution types also differed significantly between the two groups (*p* = 2.0 × 10^−224^). Patients treated at university hospitals and private hospitals were more highly represented in the TRS examination group, whereas those treated at public hospitals were more highly represented in the non‐examination group. Among the 9870 patients who underwent a TRS examination, 2819 (28.6%) were classified as having TRS. Among these 2819 patients, 1307 (46.4%) were prescribed clozapine at discharge.

**TABLE 1:** Prevalence of treatment‐resistant schizophrenia among hospitalized patients.

Variable	TRS examination (+)9870 (52.2%)	TRS examination (−)9044 (47.8%)	*p*
Age, mean (SD)	46.1 (15.6)	47.2 (16.0)	**1.1 × 10^‐6**
Sex			0.19
Male, *n* (%)	4293 (43.5%)	4020 (44.4%)	
Female, *n* (%)	5577 (56.5%)	5024 (55.6%)	
Type of facility			**2.0 × 10^‐224**
University hospital, *n* (%)	4169 (42.2%)	2518 (27.8%)	
Public hospital, *n* (%)	2561 (25.9%)	4372 (48.3%)	
Private hospital, *n* (%)	3140 (31.8%)	2154 (23.8%)	
TRS cases, *n* (%)	2819 (28.6%)	—	

*Note:* Values are presented as the mean (SD) or *n* (%). The percentages may not total 100% because of rounding. The percentage for TRS cases was calculated among patients who underwent a TRS examination (*n* = 9870). A Bonferroni correction was applied to account for multiple comparisons across three tests; *p*‐values below the Bonferroni‐corrected threshold of 0.0167 are shown in bold.

Abbreviations: SD, standard deviation; TRS, treatment‐resistant schizophrenia.

## 4 Discussion

In this study, the prevalence of TRS was determined to be 28.6% among 9870 patients with schizophrenia who underwent a TRS examination on the basis of real‐world clinical data from 205 institutions across Japan. This sample exceeded the 8044 patients included in the largest previous single study of TRS prevalence [22], making the present study the largest investigation of TRS prevalence to date. To our knowledge, this is the first study to determine the prevalence of TRS using standardized Japanese diagnostic criteria.

The TRS prevalence of 28.6% observed in the present study was lower than the pooled prevalence of 36.7% (95% confidence interval, 33.1%–40.5%) reported in a previous meta‐analysis of 50 studies comprising 29 390 patients [7]. However, the prevalence observed in the present study was nearly identical to the pooled prevalence of 28.4% reported for studies at low risk of bias in that meta‐analysis. The present study examined TRS prevalence using a large real‐world clinical dataset and standardized diagnostic criteria for TRS, thereby largely satisfying the methodological criteria used to classify studies as having a low risk of bias in the previous meta‐analysis. These methodological similarities may account for the close agreement between the prevalence observed in the present study and the pooled prevalence among studies at low risk of bias.

In the present study, 46.4% of patients with TRS were treated with clozapine. Clozapine treatment rates among patients with TRS remain below 25% in many clinical settings [23], and have been reported to be even lower in Japan than in other countries [24]. Thus, the clozapine treatment rate in the present study population may have been higher than that observed in routine clinical settings. This relatively high rate may be attributable, at least in part, to previous findings that institutions that more actively conduct TRS examinations have higher rates of clozapine prescribing [6] and that both the implementation of TRS examinations and clozapine prescribing have increased over time among EGUIDE‐participating institutions [9].

Several limitations of this study should be acknowledged. First, the study population was restricted to patients admitted to EGUIDE‐participating institutions authorized to prescribe clozapine. Therefore, caution is warranted when these findings are generalized to the overall population of patients with schizophrenia in Japan, particularly to outpatients. Second, only 52.2% of the patients underwent a TRS examination, and the distribution of institution types differed significantly between patients who did and did not undergo the examination. Previous EGUIDE studies have shown that quality indicators of treatment vary according to institution type [10]. Therefore, the TRS prevalence observed in this study may reflect the characteristics of the subgroup of patients who underwent a TRS examination.

In conclusion, on the basis of large‐scale real‐world clinical data from 205 institutions across Japan, the prevalence of TRS was determined to be 28.6% among patients with schizophrenia who underwent a TRS examination. This prevalence was nearly identical to the pooled prevalence reported for studies at low risk of bias in a previous meta‐analysis and may provide important evidence regarding the prevalence of TRS in routine clinical practice in Japan.

## Author Contributions

Y.A., T.H. and H.M. were involved in data collection and analysis and wrote the first draft of the manuscript. J.M., K.Y., T.N., H.Y., T.O., Y.Y., N.H., K.O., S.O., Y.K., K.M., H.Y., T.T., F.K., J.I., S.N., H.H. and N.Y.‐F. contributed to the interpretation of the data and data collection. K.I. and K.W. were involved in the study design and contributed to the interpretation of the data. R.H. supervised the entire project, collected the data, and was involved in the data design, analysis, and interpretation. All the authors contributed to and approved the final article.

## Funding

This study was supported by the Japan Agency for Medical Research and Development (JP16dk0307060, JP19dk0307083, and JP22dk0307112), JSPS KAKENHI (JP21K17261 and JP23K16273), Health and Labor Sciences Research (H29‐Seishin‐Ippan‐001 and 19GC1012), the Japanese Society of Neuropsychopharmacology, the Japanese Society of Mood Disorders, the Japanese Society of Clinical Neuropsychopharmacology, Shinshu Association for the Advancement of Medical Sciences, and the Japanese Society of Psychiatry and Neurology. The funders played no role in the study design, data collection and analysis, decision to publish, or manuscript preparation.

## Ethics Statement

This study was approved by the Ethics Committee of the National Center of Neurology and Psychiatry (approval number: B2022‐004) and the Ethics Committee of Dokkyo Medical University Hospital (R‐98‐4 J).

## Consent

Data were collected at each participating institution through an opt‐out consent procedure.

## Conflicts of Interest

The authors declare no conflicts of interest.

## Supporting information


**Table S1:** Requirements to become a clozapine‐licensed institution Format: Word (.docx).
**Table S2:** Definition of treatment‐resistant schizophrenia in Japan Format: Word (.docx).

## Data Availability

The data that support the findings of this study are available on request from the corresponding author. The data are not publicly available due to privacy or ethical restrictions.

## References

[1] S. Leucht , S. Siafis , J. J. McGrath , et al., “Schizophrenia,” Nature Reviews. Disease Primers 11, no. 1 (2025): 83. 10.1038/s41572-025-00667-6 41309720

[2] S. M. Couture , D. L. Penn , and D. L. Roberts , “The Functional Significance of Social Cognition in Schizophrenia: A Review,” Schizophrenia Bulletin 32, no. Suppl 1 (2006): S44–S63. 16916889 10.1093/schbul/sbl029 PMC2632537

[3] G. Remington , D. Addington , W. Honer , Z. Ismail , T. Raedler , and M. Teehan , “Guidelines for the Pharmacotherapy of Schizophrenia in Adults,” Canadian Journal of Psychiatry 62, no. 9 (2017): 604–616. 28703015 10.1177/0706743717720448 PMC5593252

[4] D. Siskind , L. McCartney , R. Goldschlager , and S. Kisely , “Clozapine v. First‐ and Second‐Generation Antipsychotics in Treatment‐Refractory Schizophrenia: Systematic Review and Meta‐Analysis,” British Journal of Psychiatry 209, no. 5 (2016): 385–392. 10.1192/bjp.bp.115.177261 27388573

[5] O. D. Howes , R. McCutcheon , O. Agid , et al., “Treatment‐Resistant Schizophrenia: Treatment Response and Resistance in Psychosis (TRRIP) Working Group Consensus Guidelines on Diagnosis and Terminology,” American Journal of Psychiatry 174, no. 3 (2017): 216–229. 27919182 10.1176/appi.ajp.2016.16050503 PMC6231547

[6] N. Yasui‐Furukori , H. Muraoka , N. Hasegawa , P. T. Tseng , M. H. Chen , and C. S. Liang , “Association Between the Examination Rate of Treatment‐Resistant Schizophrenia and the Clozapine Prescription Rate in a Nationwide Dissemination and Implementation Study,” Neuropsychopharmacology Reports 42, no. 1 (2022): 3–9. 34854260 10.1002/npr2.12218 PMC8919118

[7] E. Diniz , L. Fonseca , D. Rocha , et al., “Treatment Resistance in Schizophrenia: A Meta‐Analysis of Prevalence and Correlates,” Brazilian Journal of Psychiatry 45, no. 5 (2023): 448–458. 37718484 10.47626/1516-4446-2023-3126 PMC10894625

[8] N. Hasegawa , Y. Yasuda , N. Yasui‐Furukori , et al., “Effect of Education Regarding Treatment Guidelines for Schizophrenia and Depression on the Treatment Behavior of Psychiatrists: A Multicenter Study,” Psychiatry and Clinical Neurosciences 77, no. 10 (2023): 559–568. 37684711 10.1111/pcn.13578 PMC11488608

[9] N. Hasegawa , H. Muraoka , Y. Arai , and G. S. Rizvi , “Long‐Term Impact of EGUIDE Training on Facility‐Wide Guideline Adherence Rate in Schizophrenia and Major Depressive Disorder: A Multicenter Study,” Neuropsychopharmacology Reports 45, no. 4 (2025): e70067. 41147405 10.1002/npr2.70067 PMC12560011

[10] K. Ichihashi , H. Hori , N. Hasegawa , et al., “Prescription Patterns in Patients With Schizophrenia in Japan: First‐Quality Indicator Data From the Survey of “Effectiveness of Guidelines for Dissemination and Education in Psychiatric Treatment (EGUIDE)” Project,” Neuropsychopharmacology Reports 40, no. 3 (2020): 281–286. 32602667 10.1002/npr2.12122 PMC7722678

[11] H. Iida , J. Iga , N. Hasegawa , R. McIntyre , and H. Gill , “Unmet Needs of Patients With Major Depressive Disorder ‐ Findings From the ‘Effectiveness of Guidelines for Dissemination and Education in Psychiatric Treatment (EGUIDE)’ Project: A Nationwide Dissemination, Education, and Evaluation Study,” Psychiatry and Clinical Neurosciences 74, no. 12 (2020): 667–669. 32881226 10.1111/pcn.13143 PMC7756454

[12] N. Hashimoto , N. Yasui‐Furukori , N. Hasegawa , et al., “Characteristics of Discharge Prescriptions for Patients With Schizophrenia or Major Depressive Disorder: Real‐World Evidence From the Effectiveness of Guidelines for Dissemination and Education (EGUIDE) Psychiatric Treatment Project,” Asian Journal of Psychiatry 63 (2021): 102744. 34325252 10.1016/j.ajp.2021.102744

[13] R. Furihata , R. Otsuki , N. Hasegawa , et al., “Hypnotic Medication Use Among Inpatients With schizophrenia and Major Depressive Disorder: Results of a Nationwide Study,” Sleep Medicine 89 (2022): 23–30. 34875519 10.1016/j.sleep.2021.11.005

[14] K. Ichihashi , Y. Kyou , N. Hasegawa , et al., “The Characteristics of Patients Receiving Psychotropic Pro Re Nata Medication at Discharge for the Treatment of Schizophrenia and Major Depressive Disorder: A Nationwide Survey From the EGUIDE Project,” Asian Journal of Psychiatry 69 (2022): 103007. 35051727 10.1016/j.ajp.2022.103007

[15] H. Hori , N. Yasui‐Furukori , N. Hasegawa , et al., “Prescription of Anticholinergic Drugs in Patients With Schizophrenia: Analysis of Antipsychotic Prescription Patterns and Hospital Characteristics,” Frontiers in Psychiatry 13 (2022): 823826. 35656353 10.3389/fpsyt.2022.823826 PMC9152135

[16] Y. Kyou , N. Yasui‐Furukori , N. Hasegawa , et al., “The Characteristics of Discharge Prescriptions Including Pro Re Nata Psychotropic Medications for Patients With Schizophrenia and Major Depressive Disorder From the Survey of the “Effectiveness of Guidelines for Dissemination and Education in Psychiatric Treatment (EGUIDE)” Project,” Annals of General Psychiatry 21, no. 1 (2022): 52. 36567327 10.1186/s12991-022-00429-8 PMC9791735

[17] T. Tsuboi , Y. Takaesu , N. Hasegawa , et al., “Effects of Electroconvulsive Therapy on the Use of Anxiolytics and Sleep Medications: A Propensity Score‐Matched Analysis,” Psychiatry and Clinical Neurosciences 77, no. 1 (2023): 30–37. 36215112 10.1111/pcn.13489

[18] T. Onitsuka , T. Okada , N. Hasegawa , et al., “Combination Psychotropic Use for Schizophrenia With Long‐Acting Injectable Antipsychotics and Oral Antipsychotics: A Nationwide Real‐World Study in Japan,” Journal of Clinical Psychopharmacology 43, no. 4 (2023): 365–368. 37216369 10.1097/JCP.0000000000001704

[19] N. Hashimoto , N. Yasui‐Furukori , N. Hasegawa , et al., “Change of Prescription for Patients With Schizophrenia or Major Depressive Disorder During Admission: Real‐World Prescribing Surveys From the Effectiveness of Guidelines for Dissemination and Education Psychiatric Treatment Project,” BMC Psychiatry 23, no. 1 (2023): 473. 37380997 10.1186/s12888-023-04908-4 PMC10308621

[20] S. Ochi , H. Tagata , N. Hasegawa , et al., “Clozapine Treatment is Associated With Higher Prescription Rate of Antipsychotic Monotherapy and Lower Prescription Rate of Other Concomitant Psychotropics: A Real‐World Nationwide Study,” International Journal of Neuropsychopharmacology 25, no. 10 (2022): 818–826. 35723038 10.1093/ijnp/pyac036 PMC9593218

[21] “Medical institutions registered with the Clozaril Patient Monitoring Service [Internet]. Tokyo: The Expert Committee for Clozaril Patient Monitoring Service (Japan); updated January 2026; cited 20 January 2026,” available from, https://www.clozaril‐tekisei.jp/registered‐medical‐institutions.

[22] T. Wimberley , H. Støvring , H. J. Sørensen , H. T. Horsdal , J. H. MacCabe , and C. Gasse , “Predictors of Treatment Resistance in Patients With Schizophrenia: A Population‐Based Cohort Study,” Lancet Psychiatry 3, no. 4 (2016): 358–366. 26922475 10.1016/S2215-0366(15)00575-1

[23] J. L. Gören , A. J. Rose , E. G. Smith , and J. P. Ney , “The Business Case for Expanded Clozapine Utilization,” Psychiatric Services 67, no. 11 (2016): 1197–1205. 27301766 10.1176/appi.ps.201500507

[24] C. J. Bachmann , L. Aagaard , M. Bernardo , et al., “International Trends in Clozapine Use: A Study in 17 Countries,” Acta Psychiatrica Scandinavica 136, no. 1 (2017): 37–51. 28502099 10.1111/acps.12742

