# Supplementary material for: Prevalence of Treatment‐Resistant Schizophrenia: A Nationwide Multicenter Study
Source: Neuropsychopharmacol Rep. 2026 Sep 13;46(3):e70163. doi: 10.1002/npr2.70163 (PMC13572033; doi:10.1002/npr2.70163)
Supplement: Supplementary file 1 [file NPR2-46-e70163-s001.docx]

**Table S1. Requirements to become a clozapine-licensed Institution**

**-------------------------------------------------------------------------------------------------------------------------**

**〈Requirement 1〉**

1) On the day of blood sampling, the institution must be able to obtain laboratory test results for white blood cell count and neutrophil count, blood glucose (fasting or random), and HbA1c. (If test results reach the criteria for treatment discontinuation, daily blood testing and reporting—including on holidays—must be feasible until recovery of the values.)

2) The institution must be capable of managing neutropenia and agranulocytosis.

A system must be in place to receive prompt advice from a hematologist, and to provide treatment as needed. (Collaboration with other medical institutions is acceptable.)

If collaboration with a hematologist is difficult (including difficulty collaborating with hematologists at remote medical institutions), collaboration with a physician who is a member of the Japanese Society for Infectious Diseases, a member of the Japanese Society of Medical Oncology, or a physician deemed to have equivalent or superior experience by the Clozaril Appropriate Use Committee, and who has sufficient experience in treating agranulocytosis, is also acceptable.

The institution must be able to implement infection control measures such as administration of antibiotics and securing a private room as needed.

Emergency administration of G-CSF products must be possible (i.e., G-CSF products are stocked, or can be delivered immediately).

The institution must employ staff knowledgeable about infection control (e.g., nurses).

Antibiotics and other anti-infective agents must be kept in stock.

3) Collaboration with diabetologists must be possible (collaboration with other institutions is acceptable).

4) A personal computer with internet connectivity must be available, and eCPMS system must be operable.

**〈Requirement 2〉**

5) The institution must have at least two personnel each for: CPMS-registered physicians, Clozaril-managing pharmacists, CPMS coordination staff

(A Clozaril-managing pharmacist may also serve as CPMS coordination staff.)

6) A collaboration protocol for medical coordination in cases of agranulocytosis or glucose intolerance must be prepared.

7) The institution must pledge to comply with the “CPMS Operating Procedures

**Table S2. Definition of Treatment-resistant schizophrenia in Japan**

**--------------------------------------------------------------------------------------------**

**Treatment-resistant schizophrenia (TRS) is defined as a poor response or poor tolerability.**

**Criteria for a Poor Response**

Patients who do not respond to at least two adequate doses of antipsychotics (600 mg/day or more of chlorpromazine or an equivalent, including at least one atypical antipsychotic (risperidone, perospirone, olanzapine, quetiapine, aripiprazole, etc.)) for a sufficient period of time (at least 4 weeks), unless tolerated poorly. (c) Patients who do not respond to treatment for a sufficient period of time (more than 4 weeks). In addition, compliance with medication use should be confirmed.

a) When atypical antipsychotics are used concomitantly, the drug with the highest chlorpromazine equivalent dose should be included.

(b) For atypical antipsychotics, the patient should have been treated for at least one year.

(c) No response to treatment: The patient must have never had a condition equivalent to a GAF (Global Assessment of Functioning) score of 41 or higher.

The definition of TRS as those included in the insert package and CPMS guidance were taught in the EGUIDE guideline training, so there were no regional differences.

**Criteria for Poor Tolerance**

Patients who attempt monotherapy with two or more atypical antipsychotics, such as risperidone, perospirone, olanzapine, quetiapine, and aripiprazole, but the dose cannot be increased sufficiently to achieve a satisfactory therapeutic effect for any of the following reasons.

Patients with moderate or severe tardive dyskinesia a), tardive dystonia b), or other tardive extrapyramidal symptoms, or worsening of these symptoms.

Patients with an appearance of poorly controlled Parkinson's symptoms, akathisia, or acute dystonia.

a) A DIEPSS (Drug-Induced Extra-Pyramidal Symptoms Scale) score of 3 or higher for dyskinesia.

(b) Tardive extrapyramidal symptoms with a DIEPSS score of 3 or higher.

(c) Patients with at least one DIEPSS score of 3 or higher or two DIEPSS scores of 2 or higher, despite treatment with the upper limit of the usual dose of antiparkinsonian drugs.

(d) The presence of at least one score of 3 or more, or two scores of 2 or more, out of four items for "slow movement," "muscle stiffness," and "tremor”.

(d) A score of 3 or more for akathisia on the DIEPSS despite various treatments, including antiparkinsonian medication at the upper limit of the usual dosage.

(e) Frequent acute dystonia corresponding to a DIEPSS "dystonia" score of 3 despite various treatments, including the administration of antiparkinsonian drugs at the upper limit of the usual dose, causing great distress to the patient
